# Enhanced Antiviral Function of Magnesium Chloride-Modified Heparin on a Broad Spectrum of Viruses

**DOI:** 10.3390/ijms221810075

**Published:** 2021-09-17

**Authors:** Kemal Mese, Oskar Bunz, Wolfram Volkwein, Sahithya P. B. Vemulapalli, Wenli Zhang, Sebastian Schellhorn, Kristin Heenemann, Antje Rueckner, Andreas Sing, Thomas W. Vahlenkamp, Anna-Lena Severing, Jian Gao, Malik Aydin, Dominik Jung, Hagen S. Bachmann, Kurt S. Zänker, Ulrich Busch, Armin Baiker, Christian Griesinger, Anja Ehrhardt

**Affiliations:** 1 Virology and Microbiology, Center for Biomedical Education and Research (ZBAF), Witten/Herdecke University, 58453 Witten, Germany; Kemal.Mese@uni-wh.de (K.M.); Oskar.Bunz@uni-wh.de (O.B.); Wenli.Zhang@uni-wh.de (W.Z.); Sebastian.Schellhorn@uni-wh.de (S.S.); Jian.Gao@uni-wh.de (J.G.); Armin.Baiker@lgl.bayern.de (A.B.); 2 Department of Prosthodontics, School of Dentistry, Faculty of Health, Witten/Herdecke University, 58455 Witten, Germany; 3 Bavarian Health and Food Safety Authority (LGL), 85764 Oberschleissheim, Germany; Wolfram.Volkwein@lgl.bayern.de (W.V.); Andreas.Sing@lgl.bayern.de (A.S.); Ulrich.Busch@lgl.bayern.de (U.B.); 4 Department of NMR-Based Structural Biology, Max Planck Institute for Biophysical Chemistry, 37077 Göttingen, Germany; save@nmr.mpibpc.mpg.de; 5 Center for Infectious Diseases, Institute of Virology, Faculty of Veterinary Medicine, University of Leipzig, 04103 Leipzig, Germany; Kristin.Heenemann@vetmed.uni-leipzig.de (K.H.); antje.rueckner@sanktgeorg.de (A.R.); thomas.vahlenkamp@uni-leipzig.de (T.W.V.); 6 Centre for Biomedical Education and Research (ZBAF), Institute for Translational Wound Research, Witten/Herdecke University, 58453 Witten, Germany; Anna-Lena.Severing@uni-wh.de; 7 Center for Child and Adolescent Medicine, Center for Clinical and Translational Research (CCTR), Helios University Hospital Wuppertal, Witten/Herdecke University, 42283 Wuppertal, Germany; Malik.Aydin@uni-wh.de; 8 Centre for Biomedical Education and Research, Institute of Pharmacology and Toxicology, Witten/Herdecke University, 58453 Witten, Germany; Dominik.Jung@uni-wh.de (D.J.); Hagen.Bachmann@uni-wh.de (H.S.B.); 9 Center for Biomedical Education and Research (ZBAF), Institute of Immunology, Witten/Herdecke University, 58453 Witten, Germany; Kurt.Zaenker@uni-wh.de

**Keywords:** modified heparin, magnesium chloride, antiviral, HSV-1, adenovirus, SARS-CoV-2, NMR

## Abstract

Previous studies reported on the broad-spectrum antiviral function of heparin. Here we investigated the antiviral function of magnesium-modified heparin and found that modified heparin displayed a significantly enhanced antiviral function against human adenovirus (HAdV) in immortalized and primary cells. Nuclear magnetic resonance analyses revealed a conformational change of heparin when complexed with magnesium. To broadly explore this discovery, we tested the antiviral function of modified heparin against herpes simplex virus type 1 (HSV-1) and found that the replication of HSV-1 was even further decreased compared to aciclovir. Moreover, we investigated the antiviral effect against the new severe acute respiratory syndrome coronavirus type 2 (SARS-CoV-2) and measured a 55-fold decreased viral load in the supernatant of infected cells associated with a 38-fold decrease in virus growth. The advantage of our modified heparin is an increased antiviral effect compared to regular heparin.

## 1. Introduction

Viruses are a serious threat to humans and livestock. There are well-characterized viruses causing infectious diseases, but also emerging viruses play an increasingly important role in a globalized world. In many cases, there are no adequate therapies available against most infectious diseases, largely because viruses provide few targets for therapeutic treatment. Recently, coronavirus disease 2019 (COVID-19) caused by the novel coronavirus SARS-CoV-2 started in China [1] and spread across the globe. Virus infections for which no drug or vaccine is available, demand the urgent development of therapeutics. Well-known antiviral medications, for example against the human immunodeficiency virus (HIV) or against herpes viral disease, target virus-specific sites of the uptake or replication cycle. These drugs are therefore not necessarily transferable to other virus families. From a clinical point of view, a broad-spectrum therapy against as many virus species as possible is highly desirable. 

A possible broad-spectrum antiviral treatment could be the already known drug heparin. There is evidence that heparin displays antiviral activity against a variety of different viruses as for instance feline immunodeficiency virus FIV [2], HIV [3] and Zika virus [4]. In the case of the SARS-CoV-2 pandemic, various benefits of heparin for the treatment of COVID-19 patients were already reported and a better outcome of patients undergoing treatment with low molecular weight heparin is known. So far, it has been attributed to the anti-thrombogenic [5,6,7,8] and anti-inflammatory effect [9] of heparin. 

However, the antiviral effect of heparin has not been studied thoroughly in vivo. This may be due to the rather moderate reduction in viral load achieved by heparin and to the expected undesirable anticoagulant effect. To increase the antiviral effect of heparin, we performed experiments with the combination of heparin and divalent cations such as magnesium chloride. We initially observed a robust antiviral effect of this modified heparin on human adenovirus type 5 (HAdV5) predominantly responsible for respiratory tract infections. To broadly explore this observation, further experiments with herpes simplex virus type 1 (HSV-1) and SARS-CoV-2 were performed. The results were in concordance with effects observed for adenovirus. We consider that after further investigation of the underlying mechanisms, modified heparin could be developed as a valuable antiviral treatment option.

## 2. Results

**Figure 1:**
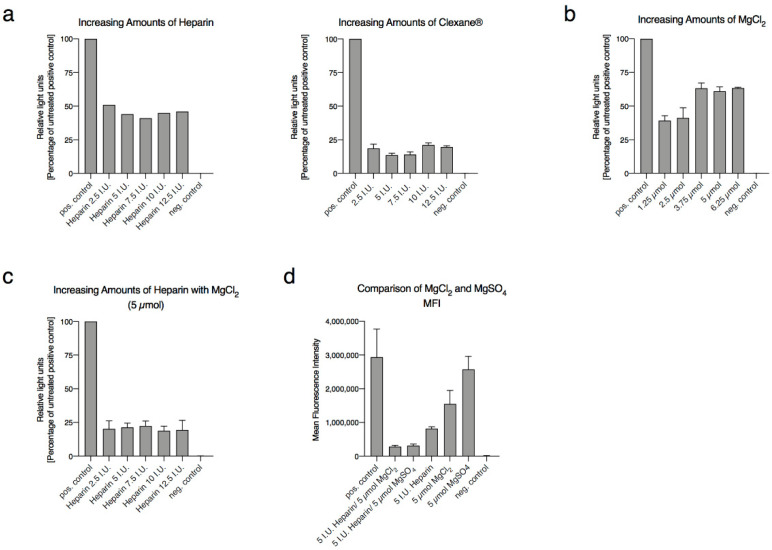
The effects of different concentrations of heparin, enoxaparin and MgCl_2_/MgSO_4_ on human adenovirus type 5 (HAdV5). Virus and further components were mixed pre-transduction and added to CHO-K1 cells. The luciferase measurements were performed 26 h post-transduction. All experiments included a positive control referring to untreated HAdV5: (**a**) CHO-K1 cells were infected with 1000 viral particles per cell with HAdV5 in the presence of different international units (I.U.) of heparin and enoxaparin; (**b**) Transduction rates of HAdV5 in the presence of increasing amounts of MgCl_2_; (**c**) Infectivity of HAdV5 at 1000 viral particles per cell in the presence of different amounts of heparin and 5 µmol of MgCl_2_; (**d**) Effects of MgCl_2_ and MgSO_4_ on HAdV5 transduction rates. Experiments were performed in triplicates and repeated three times. The mean standard errors are shown.

We first compared the antiviral effect against human adenoviruses of low-molecular-weight heparin and unfractionated heparin (high-molecular-weight heparin). Low-molecular-weight heparins such as enoxaparin bound to antithrombin III preferentially inhibit coagulation factor Xa [10,11], whereas unfractionated heparin inhibits coagulation factors IIa and Xa [12]. Both heparin forms resulted in virus transduction inhibition in CHO-K1 cells after transduction with the HAdV5-based vector (Figure 1a). For further experiments, we decided to use unfractionated heparin, because the use of this heparin is established in the clinic for subcutaneous and intravenous application [13,14], is easy to monitor [15,16] and shows improved antagonizability using protamine sulfate [17]. In contrast, low-molecular-weight heparin is only approved for subcutaneous application. 

### 2.1. Magnesium Enhances the Antiviral Function of Heparin

It was described in previous studies that the binding of heparin to different metals resulted in a conformational change and that this can reduce the inhibitory function of heparin in the coagulation cascade and therefore modulate the activity of heparin [18,19,20,21,22,23]. Moreover, it was found that Ca^2+^ ions show a stronger affinity to heparin than Mg^2+^ ions [20], yet, Mg^2+^ but not Ca^2+^ can be applied clinically without side effects [24]. Therefore, we modified heparin with magnesium chloride (MgCl_2_). To explore the antiviral function of modified heparin, we initially tested different concentrations of MgCl_2_ and heparin and their inhibitory effect on human HAdV5 causing respiratory disease and HAdV type 50 (HAdV50). Both naturally occurring viruses were augmented [25,26] containing an expression cassette consisting of monocistronic luciferase and green fluorescent protein (GFP). Therefore, they are ideal to analyze whether modified heparin inhibits virus transduction efficiencies. After optimizing and testing different MgCl_2_ concentrations, we decided to utilize a concentration of 5 µmol for the following experiments (Figure 1b). In a further step, we optimized the formulation for MgCl_2_-modified heparin by keeping the MgCl_2_ concentration constant. The optimal stoichiometry of the heparin–magnesium chloride formulation was 5 I.U. of heparin and 5 µmol MgCl_2_ (Figure 1c). In the following experiments, we refer to this optimized heparin–magnesium chloride formulation as modified heparin. To exclude the involvement of chloride potentially contributing to the strong antiviral function, we compared the enhanced antiviral effect of heparin either modified with 5 µmol MgCl_2_ or 5 µmol MgSO_4_ (Figure 1d).

### 2.2. Nuclear Magnetic Resonance Analyses Reveal Conformational Change of Iduronic Acid of Heparin When Complexed with Magnesium Chloride or Calcium Chloride

**Figure 2:**
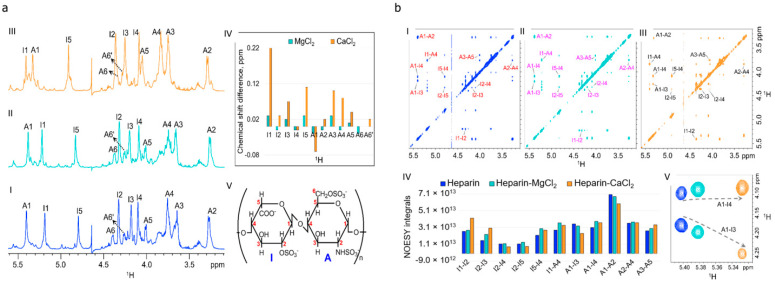
NMR analyses of magnesium chloride modified heparin: (**a**) Comparison of a portion of 1D ^1^H NMR spectra of heparin (I), heparin–Mg^2+^ (II), and heparin–Ca^2+^ (III) acquired in 99.95% D_2_O at 37 °C. Proton chemical shift assignment of iduronic acid, and glucosamine, residues are shown on top of each resonance. (IV) Bar plot showing ^1^H chemical shift perturbation of heparin in the presence of Mg^2+^ and Ca^2+^. ^1^H chemical shift difference between heparin–M^2+^ (M = Mg, Ca) and heparin are presented on the y-axis. Positive value indicates the deshielding of respective protons in the presence of M^2+^ (M = Mg, Ca). (V) Structure of the repeating disaccharide (iduronic acid-glucosamine) unit of heparin; (**b**) A portion of 2D NOESY spectra of heparin (I), heparin–Mg^2+^ (II), and heparin–Ca^2+^ (III) in 99.95% D_2_O at 37 °C. Assignments are shown for the characteristic NOE cross-peaks. (IV) Bar plot representation of intra- and inter-residue NOE cross peak volumes. (V) Change in NOESY cross peak intensities of inter-residue A1-I4 and A1-I3 protons of heparin (blue), heparin–Mg^2+^ (cyan), and heparin–Ca^2+^ (orange).

Next, we further characterized the modified heparin by performing nuclear magnetic resonance (NMR) analyses of MgCl_2_ bound to heparin. As a reference with a higher affinity to heparin, we also investigated CaCl_2_ bound to heparin. Chemical shift assignment of the disaccharide (Iduronic acid, **I** and Glucosamine, **A**) subunit of heparin and heparin–M^2+^ (M = Mg and Ca) was carried out using 2D DQF-COSY (Figure S1a) spectra acquired in 99.95% D_2_O at 37 °C. Two coupled spin networks were identified with 5 and 7 protons for iduronic acid and glucosamine residues, respectively. Significant changes were observed in ^1^H chemical shifts of heparin upon coordinating Mg^2+^ and Ca^2+^ (Figure 2a), however, changes caused by binding of Mg^2+^ were relatively small, which is in agreement with the previously reported measurements [27]. Observed changes in the ^1^H chemical shifts of iduronic acid and glucosamine suggest that both the residues of the disaccharide subunit of heparin are involved in Mg^2+^ and Ca^2+^ complexation. ^3^*J_HH_* couplings are valuable NMR structural parameters for understanding the intra-residue conformational dynamics of the disaccharide subunit of heparin [28] upon complexation with Mg^2+^ and Ca^2+^. However, the unambiguous determination of ^3^*J_HH_* couplings is hampered due to the broad lines observed in the ^1^H NMR spectrum of heparin. In order to overcome this issue, we employed a procedure DISCONOE (differences and sums of traces from DQF-COSY and NOESY) which is the modified procedure of the DISCO [29] method described by Kessler and co-workers, for extracting ^3^*J_HH_* couplings from broad signals (Figure S1b). The observed decrease in ^3^*J_HH_* couplings of I1–I2, I2–I3, and I4–I5 (Table S1) of iduronic acid suggests that the conformational equilibrium between ^1^C_4_ and ^2^S_O_ [30] shifts towards the chair conformation due to metal ion binding to heparin. Nevertheless, the three-bond proton–proton couplings of heparin in the presence of Mg^2+^ are in between that observed for heparin and heparin–Ca^2+^. Iduronic acid conformer (^1^C_4_:^2^S_O_) populations of 67:33, 69:31, and 83:17 for heparin, heparin–Mg^2+^, and heparin–Ca^2+^, respectively, were obtained from the linear least-squares fitting of the experimental and calculated ^3^*J_HH_* couplings. Opposite to iduronic acid, metal ion binding to heparin causes relatively negligible changes in ^3^*J_HH_* couplings of glucosamine indicating that the glucosamine residue exists predominantly in the ^4^C_1_ chair conformation irrespective of the presence or absence of the divalent metal ions, which is further supported by the observed NOE correlations. The increase in intra-residue I1–I2, I2–I3, and I5–I4 and inter-residue I1-A4 NOE cross peak integral (Figure 2b) of heparin upon binding Mg^2+^ and Ca^2+^ indicate the increase in ^1^C_4_ conformer population of iduronic acid, which is in line with the conclusions drawn from the ^3^*J_HH_* couplings. The populations of ^1^C_4_ and ^2^S_O_ conformers of Iduronic acid derived from the linear least-squares fitting of the experimental and calculated (please see methods section) NOE cross peak volumes are 54:46, 60:40, and 75:25 for heparin, heparin–Mg^2+^, and heparin–Ca^2+^, respectively, in agreement with the trend in conformational changes of iduronic acid derived from the ^3^*J_HH_* analysis. The average of ^1^C_4_:^2^S_O_ populations of iduronic acid obtained from the combined NOESY/^3^*J_HH_* analysis are 60:40, 64:36, and 79:21 for heparin, heparin–Mg^2+^, and heparin–Ca^2+^, respectively. The results on the comparison of heparin with those reported by Ferro and colleagues [31]. Furthermore, the increase in A1-I4 and decrease in A1-I3 NOE cross peak intensities of heparin–M^2+^ (M = Mg, Ca) compared to the free heparin suggest that the conformation of A-I glycosidic linkage altered in the presence of divalent ions to facilitate the complexation of negatively charged COO^−^ and OSO^3−^ groups on the adjacent residues to Mg^2+^ and Ca^2+^.

### 2.3. Antiviral Function of Modified Heparin Is Not Cell-Type Dependent

**Figure 3:**
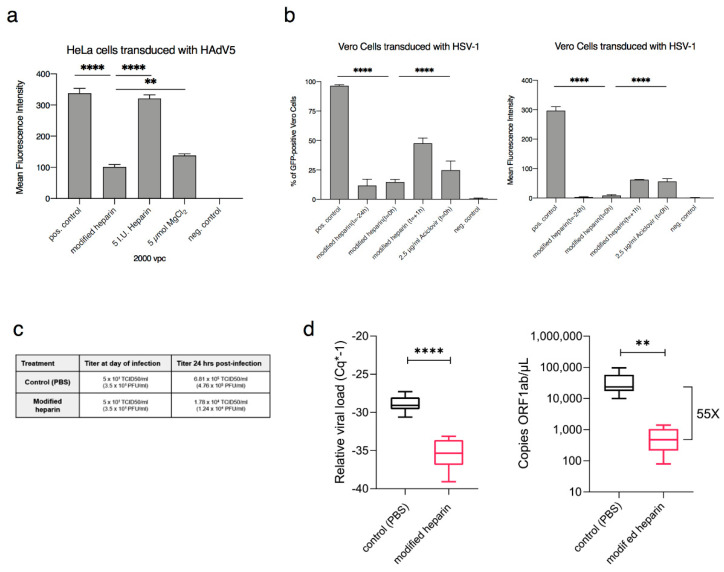
Antiviral effect of modified heparin: (**a**) Modified heparin inhibits virus transduction of human adenovirus 5 (HAdV5) in HeLa cells. The mean fluorescent intensity (MFI) shows significantly lower transduction efficiencies after treatment with modified heparin compared to untreated control. For statistical analyses one-way ANOVA was performed, displayed are means + standard deviation. ** *p*-values ≤ 0.005; **** *p*-values ≤ 0.00005; (**b**) Measurements of herpes simplex virus-1 (HSV-1) on Vero cells showed decreased transduction efficiencies after treatment with heparin/MgCl_2_ at different time points: *t* = −24 h (24 h before transduction), *t* = 0 h (at the time point of transduction) and *t* = +1 h (1 h after transduction). As control we compared the results with aciclovir treated cells (*t* = 0 h). Percentage of GFP-positive cells and mean fluorescent intensity (MFI) are displayed. For statistical analyses one-way ANOVA was performed, displayed are means + standard deviation. **** *p*-values ≤ 0.00005. pos. control: untreated virus; neg. control: cells without virus; RLU: relative light units; vpc: viral particle numbers per cell; (**c**) Antiviral effect against SARS-CoV-2. Viral load in vitro in Vero E6 cells 24 h post-transduction with SARS-CoV-2 in the presence or absence of magnesium-modified heparin. Vero E6 cells supplemented either with or without modified heparin were infected with 5 × 101 TCID50 of SARS-CoV2 per well (day 0); (**d**) Viral load in the cell culture supernatants analyzed by RT-qPCR (left panel) and RT-ddPCR (right panel) 24 h post-transduction. Supernatants of infected Vero E6 cells from experiment c, cultivated in the presence/absence of magnesium-modified heparin were collected and subjected to RT-qPCR and RT-ddPCR analysis. Experiments are from *n* = 10 biological replicates. Evaluation and graphical representation were carried out with GraphPad (Version 8.3.1, San Diego, CA, USA), *p*-values from student *t*-test (equal distribution, two-sided): **** *p* < 0.00005 and ** *p* < 0.005.

Next, we performed virus dose-escalation studies and we analyzed the sequential application of MgCl_2_-modified heparin and virus. We found that the modified heparin-based antiviral function is virus-dose dependent and that it should either be applied one hour before virus transduction or simultaneously with virus transduction (Figure S2a,b). Further experiments were performed in HeLa and A549 cells and we observed a significant decrease in virus transduction rates (Figure 3a and Figure S3b). In addition, we transduced modified heparin-treated primary human cells such as primary fibroblasts (Figure S3a) with HAdV5 and observed an antiviral function in both cell types.

### 2.4. Antiviral Function of Modified Heparin Is Based on Unspecific Shielding of Virus Surface Proteins

To understand the interaction between heparin and HAdV we performed human coagulation factor X (hFX) competition assays to explore the hexon binding ability of heparin. For this purpose, we used human adenovirus type 50 (HAdV50) because HAdV50 is known to display high binding affinities to hFX with important implications for adenovirus tropism [32]. Moreover, the binding of hFX to hexon refers to enhanced virus uptake into coxsackievirus and adenovirus receptor (CAR)-negative SKOV3 cells. Here we found that the binding affinity of modified heparin to HAdV50 virions seemed to be stronger than the binding of HAdV50 to hFX (Figure S4a). Further experiments were performed to explore adenovirus transduction in CHO-K1 cells stably expressing the major adenovirus receptors CD46 and CAR. Cells were infected with HAdV5, which is known to bind via fiber knob to CAR [33,34] and 26 h post-transduction luciferase measurements were performed. Obtained results revealed significant inhibition of virus cell entry into target cells for both cell lines transduced with treated virus (Figure S4b) hinting towards a potential covering effect of the cell surface or the viral surface proteins by modified heparin. This observation was further explored by ELISA performed with intravenous immunoglobulin (IVIG). ELISA experiments revealed that the binding of IVIG to the virus was decreased by modified heparin (Figure S4c).

### 2.5. Modified Heparin Shows an Antiviral Function on HSV-1 and SARS-CoV-2

Since adenoviruses are non-enveloped viruses, we addressed the question of whether the enhanced antiviral function of modified heparin can also be translated to enveloped viruses. For this purpose, we performed experiments using GFP-tagged HSV-1. For HSV-1 the antiviral effect of modified heparin was compared with aciclovir, a drug commonly used against HSV-1 infections. As shown in Figure 3b the enhanced antiviral function of modified heparin against HSV-1 transduction was even more efficient than aciclovir. To assure that the decrease in viral transduction on tested cells is not caused by cell death mediated by treatment with modified heparin, we performed an Annexin V assay and BCL2 activation staining on Vero cells and could exclude cell death caused by the antiviral drug (Figure S5a,b).

SARS-CoV-2 belongs to the coronavirus family and represents an enveloped virus. Here we tested the increased antiviral function of modified heparin using a SARS-CoV-2 infected COVID-19 patient isolate. For virus rescue and amplification, we applied Vero E6 cells. The virus titer in the cell supernatant was determined and compared to the results of the RT-qPCR and RT-ddPCR measured titers. As shown in Figure 3c,d we found a strong antiviral effect on SARS-CoV-2. Viral growth was decreased 38-fold 24 h post-infection after a single treatment with our modified heparin (Figure 3c) and at the same time, the viral load was decreased 55-fold in the supernatant of infected cells as measurements indicate using RT-qPCR and RT-ddPCR (Figure 3d). Our results are in concordance with a recently published study showing that infection of SARS-CoV-2 is decreased by modulated cellular heparan sulfate [35]. Interestingly, another recent study [36] has demonstrated that cell attachment of SARS CoV2 depends on the expression of heparan sulfate and syndecan-1, a proteoglycan decorated with heparin sulfate molecules. Therefore, it can be speculated, that the added heparin/magnesium complex effectively competes with the cellular heparin sulfate preventing effective binding of the virus.

## 3. Discussion

In several studies, the antiviral effect of heparin has already been examined and proven [37,38,39]. However, because of an unfavorable relation of the heparin antiviral effect and unintended bleeding events, this is of limited clinical use. The results of our study demonstrate that heparin in the presence of magnesium chloride [18,20,21] leads to an enhanced antiviral function. This is accompanied by a conformational change induced by the presence of Mg^2+^ depopulating the conformation that is believed to be responsible for the anti-coagulation effect of heparin.

Our results indicate an enhanced antiviral function of modified heparin based on the interaction of heparin and magnesium. The achieved results show no differences between magnesium sulfate and magnesium chloride modified heparin regarding the antiviral effect. Interestingly, comparing solely magnesium chloride and magnesium sulfate on the transduction rate a difference is detectable.

Experiments aiming to discover the changes in the binding capacity of virus surface proteins (exemplary on adenoviruses) revealed that the effect of modified heparin is not based on the viral protein charge itself. For example, our results hint towards the hypothesis that our modified heparin decreases the binding capacity of adenoviral proteins [40,41], towards human coagulation factor X. Furthermore, we found decreased antibody detection of HAdV by IVIG (shown via ELISA), which may strengthen our hypothesis.

Recently, publications on the treatment of COVID-19 patients with heparin have increased. The multi-target function of heparin includes not only anticoagulant and anti-inflammatory but also direct antiviral effects [35,42,43,44,45,46,47]. Our studies on SARS-CoV-2 are in concordance with the previously published dose-dependent reduction of SARS-CoV-2 plaques caused by heparin treatment in vitro [46]. In contrast, we showed the reduced transduction and replication of SARS-CoV-2 by RT-qPCR.

In summary, here we show a highly non-specific transduction inhibition on a broad spectrum of viruses including enveloped SARS-CoV-2 and HSV-1 viruses and non-enveloped adenovirus.

## 4. Materials and Methods

### 4.1. Optimization of the Antiviral Effect of Modified Heparin on Eukaryotic Cells

HeLa cells, A549 cells and CHO-K1 cells were cultured in DMEM supplemented with 10% fetal bovine serum (FBS) and 1% penicillin/streptomycin (all PAN-Biotech GmbH, Aidenbach, Germany) under humidified conditions with 5% CO_2_ at 37 °C. For details regarding the optimization of the experiments, please refer to the supplementary methods section.

### 4.2. Antiviral Effect of Modified Heparin on Primary Human Cells

Human dermal fibroblasts were isolated from skin tissue of patients undergoing abdominoplasty. The relevant ethical approval for usage of human abdominal skin was obtained by the local ethics committee (No. 84/2017). For cleaning and disinfection, the skin was washed in 70% ethanol, followed by rinsing three times in PBS containing 1% penicillin/streptomycin (both PAN-Biotech GmbH, Aidenbach, Germany). After removing the subcutaneous fatty tissue, the skin was cut into thin strips (0.5 × 3 cm), covered with thermolysin (Sigma-Aldrich, St. Louis, MO, USA) solution (0.5 mg/mL in PBS) and incubated overnight at 4 °C. Next, the epidermal layer could be removed and the dermis was chopped into very small pieces (5 mm²) which were incubated in collagenase solution (33 U/mL in PBS, Biochrom GmbH, Berlin, Germany) for 3 h at 37 °C. Thereafter, the obtained suspension was purified through a cell strainer (70 µm) and centrifuged at 110× *g* for 10 min. The resulted pellet was resuspended in culture medium and isolated cells were seeded into culture flasks at a density of 4 × 10^4^ cells/cm². Human dermal fibroblasts were cultured in DMEM supplemented with 10% fetal bovine serum (FBS) and 1% penicillin/streptomycin (all PAN-Biotech GmbH, Aidenbach, Germany) under humidified conditions with 5% CO_2_ at 37 °C. After reaching a confluence of about 80–90%, cells were frozen in DMEM supplemented with 20% FBS, 10% dimethyl sulfoxide (DMSO, Sigma-Aldrich, St. Louis, MO, USA) and 1% penicillin/streptomycin to 2 × 10^6^ cells/vial and stored at liquid nitrogen.

The preparation of HAdV5 and HAdV50 was performed as described previously [26]. We diluted HAdVs for the individual experiments and applied respective virus particle numbers per cell (vpc). We applied 23 vpc for primary human fibroblasts and 2.5 vpc for primary human epithelial cells. In this experimental setup, untreated viruses served as positive control and the combination treatment of heparin (5 I.U.) and magnesium chloride (5 µmol) and heparin and magnesium chloride only were compared. The transduction efficiencies of tagged HAdV5 in primary cells with and without treatment were measured by determining the reporter gene (luciferase) expression levels 26 h post-transduction via luciferase assay. In brief, 50 µL medium of each well (96 well plate) were mixed with 50 µL of Nano-Glo substrate (Promega GmbH, Mannheim, Germany). The luminescence was detected using a plate reader (Tecan Group Ltd., Männedorf, Switzerland).

### 4.3. Formulation of Modified Heparin

Heparin and enoxaparin as low molecular weight heparin were purchased from Ratiopharm (Ulm, Germany) at a concentration of 50.000 I.U./mL. MgCl_2_, CaCl_2_ and MgSO_4_ were obtained from Carl Roth (Dautphetal-Buchenau, Germany). If not stated otherwise a concentration of 5 µmol/L and 5 I.U. of MgCl_2_ and heparin, respectively, were applied.

### 4.4. NMR Analyses

NMR spectra were acquired on Bruker Avance NEO 800 MHz and Bruker Avance III HD 600 MHz spectrometers equipped with 5 mm cryoprobes. All the spectra were measured in 99.95% D_2_O at 37 °C and referenced to external DSS (2,2-dimethyl-2-silapentane-5-sulfonate) standard. A recycle delay of 2 s was used in all the experiments. Then, 2D-DQF-COSY and NOESY spectra were acquired with a spectral width of 4166 Hz in both the direct and indirect dimensions and the matrix size of 8192 (F1) × 512 (F2) points. Then, 2D NOESY spectra were recorded with a 200 ms mixing time. NMR spectra were processed using Topspin 4.0.8 (Bruker Biospin, Ettlingen, Germany), NMRpipe [48] and analyzed by using Sparky [49]. DISCONOE procedure: For AX spin system, the addition and subtraction of traces of in-phase cross-peak from 2D-NOESY and anti-phase cross-peak from 2D-DQF-COSY spectra of respective proton pair result in an in-phase doublet. DFT calculations were performed with Gaussian 09 [50]. ^3^*J_HH_* couplings for iduronic acid in ^1^C_4_ and ^2^S_O_ conformations were computed using NMR = giao, spin-spin [48] method with mpw1pw91 [51] functional and 6-311++g(2d,p) basis set. The calculated ^1^H-^1^H internuclear distances of repeating disaccharide unit of heparin with iduronic acid in ^1^C_4_ and ^2^S_O_ conformations and glucosamine in ^4^C_1_ conformation (the solution NMR structure of heparin, PDB ID: 1 hpn) [52], were converted into NOE cross peak volumes using A1-A2 (internuclear distance: 2.55 Å and observed NOE cross peak volume: 3.68 × 10^13^) protons of glucosamine as a reference.

### 4.5. Modified Heparin Tested on HSV-1

For HSV-1 virus production, a previously described bacterial artificial chromosome (BAC) containing the HSV-1 genome was used to rescue the GFP tagged virus as previously described [53] and stored as a stock at −80 °C. The HSV-1 transduction efficiency was tested with incrementally increasing virus dosages and measured via flow cytometry analyses (Navios Flow Cytometer, Beckmann Coulter, Krefeld, Germany). Vero cells were cultured in Dulbecco’s Modified Eagle’s Medium (DMEM, PAN-Biotech GmbH, Aidenbach, Germany) supplemented with 10% fetal bovine serum (FBS, PAN-Biotech GmbH, Aidenbach, Germany) and 1% penicillin/streptomycin (PAN-Biotech GmbH, Aidenbach, Germany). Cells were counted and 1 × 10^5^ cells were seeded per 24-well plate.

An optimal virus concentration leading to over 80% GFP-positive Vero cells after 24 h was used for the following experiments. In this experimental setup, different time points of transduction were examined and compared to the untreated positive control. Heparin (5 I.U.)/MgCl_2_ (5 µmol) was applied in different time points: by seeding the cells (*t* = −24), direct after transduction (*t* = 0) and one hour after transduction (*t* = +1). As control we compared the results with aciclovir treated cells (*t* = 0 h). After 24 h incubation cells were harvested, washed two times with PBS (PAN-Biotech GmbH, Aidenbach, Germany) and evaluated via flow cytometry. GFP-positive cells and the mean fluorescence intensity (MFI) were analyzed. Statistical differences were determined using the one-way analysis of variance (ANOVA) test (alpha = 0.05) referring to the positive control (GraphPad Software, Version 8.3.1, San Diego, CA, USA).

### 4.6. SARS-CoV-2 Isolation, Infection and Detection

SARS-CoV-2 isolate LGL-SCoV2-I1 was isolated from a patient with laboratory-confirmed diagnosis of SARS-CoV-2 infection as described recently [54]. Briefly, the patient’s throat swab sample was filtered through a 0.45 µm Minisart^®^ syringe filter (Sartorius Stedim Biotech Inc., Goettingen, Germany) and inoculated on a monolayer of Vero E6 cells (ATCC^®^ CRL-1586™) for five days at 37 °C in a 5% carbon dioxide atmosphere until typical cytopathic effect (CPE) was visible. Vero E6 cells were grown in DMEM growth media supplemented with 10% heat-inactivated fetal calf serum, 1% penicillin–streptomycin solution (10,000 U/mL, Gibco, Invitrogen Inc., Carlsbad, CA, USA) and 1% Fungizone (250 µg/mL, Gibco, Invitrogen Inc., Carlsbad, CA, USA). After verifying the integrity of a SARS-CoV-2 isolate in the cell culture supernatant by RT-qPCR utilizing the RealStar^®^ SARS-CoV-2 RT-PCR Kit 1.0 (Altona Diagnostics Inc., Hamburg, Germany), the isolate LGL-SCoV2-I1 was amplified in Vero E6 cells. Viral stocks were generated from infected cell culture supernatants 36 h post-infection and stored at −80 °C until further usage. The Tissue Culture Infection Dose 50 (TCID50) per ml of viral stock was determined by serial dilution assay. Briefly, 10-fold serial dilutions of the viral stock were generated and Vero E6 cells inoculated in duplicates until CPE was visible. The TCID50/mL was calculated utilizing the TCID50_calculator_v2_17-01-20_MB online tool (https://www.klinikum.uni-heidelberg.de/zentrum-fuer-infektiologie/molecular-virology/welcome/downloads, accessed on 14 September 2021).

In order to assay putative antiviral effects of modified heparin, viral growth was measured 24 h post-infection on Vero E6 cells in the presence and absence of the substance. For this purpose, Vero E6 cells were supplemented with heparin (5 I.U.)/MgCl_2_ (5 µmol) one hour prior to viral infection and incubated at 37 °C in a 5% carbon dioxide atmosphere. As a negative control, PBS was included. After pre-incubation of the Vero E6 cells with modified heparin or PBS as control, 5 × 10^1^ TCID50/mL of SARS-CoV-2 was added to the cells and they were further incubated at 37 °C in a 5% carbon dioxide atmosphere. All experiments were performed in 10 biological replicates (*n* = 10) in 24 well culture plates (Greiner, BIO-ONE Inc., Frickenhausen, Germany). Infectious viral titers (in TCID50/mL) were determined by serial dilution assay as described above. Furthermore, on each day 140 µL cell culture supernatant was aliquoted, heat-inactivated at 60 °C for one hour and stored at −20 °C till RNA isolation.

### 4.7. RNA Isolation and RT-qPCR of SARS-CoV-2

Viral RNA from SARS-CoV-2 infected Vero E6 cells was isolated from 140 µL cell culture supernatant using the QIAamp Viral RNA Mini Kit (Qiagen, Hilden, Germany) according to manufacturer’s instructions. After RNA isolation the samples were stored at −80 °C until further use. Prior quantitative reverse transcription PCR (RT-qPCR) samples were diluted 1:1000 in nuclease-free water since heparin is a known PCR inhibitor [55,56,57,58]. Additionally, and to exclude PCR inhibition by modified heparin, an internal PCR control was added. To detect SARS-CoV-2 RNA by RT-qPCR, the FTD SARS-CoV-2 kit (Fast Track Diagnostic, Sliema, Malta) was used according to the instructions of the manufacturer. The RT-qPCR reaction itself was performed within a BioRad CFX real-time PCR detection system. For the data analysis, the Bio-Rad CFX Maestro 1.1 software version 4.1.2433.1219 was used.

### 4.8. RT-ddPCR

To determine the SARS-CoV-2 copy number in cell culture supernatants, reverse transcription droplet digital PCR (RT-ddPCR) targeting the ORF1ab gene of SARS-CoV-2 was used. The primers ORF1ab forward (5′-CCCTGTGGGTTTTACACTTAA-3′) and ORF1ab reverse (5′-ACGATTGTGCATCAGCTGA-3′) published by the Chinese Center for Disease Control and Prevention (CCDC) were used [59,60]. Additionally, the probe from the CCDC targeting ORF1ab was used with the slight modification that the Black Hole Quencher 1 (BHQ^®^-1) was replaced by a ZEN/Iowa Black FQ double quencher (5′-/56-FAM/CCGTCTGCG/ZEN/GTATGTGGA AAGGTTATGG/3IABkFQ/-3). Primers and probes were purchased from Integrated DNA Technologies (IDT, Leuven, Belgium). The RT-ddPCR reaction mix was set up with a total volume of 20 µL. This mix contained 10 µL 2× One-Step RT-ddPCR Advanced Kit for probes (Bio-Rad, Munich, Germany), 400 units of reverse transcriptase (Bio-Rad, Munich, Germany), 15 mM dithiothreitol (DTT) (Bio-Rad, Munich, Germany) 400 nM of primers ORF1ab forward and ORF1ab reverse, 100 nM probe and 2 µL of isolated RNA from cell culture supernatants diluted 1:100 or 1:1000 in nuclease-free water. After the RT-ddPCR reaction mixture was prepared, droplets were generated using a Q × 200 drop generator according to the instructions of the manufacturer (Bio-Rad, Munich, Germany). Afterward, the generated droplets were subjected to PCR with the following thermal cycling conditions: reverse transcription at 45 °C for 60 min, enzyme activation at 95 °C for 10 min, 60 cycles of 95 °C for 30 s and 59 °C for 2 min, and finally for enzyme deactivation 98 °C for 10 min. For all steps, a ramp rate of 1 °C/s was used. After amplification, fluorescent and non-fluorescent droplets were analyzed and counted using the Q × 100 Droplet Reader (Bio-Rad, Munich, Germany) in combination with QuantaSoft software (Bio-Rad, Munich, Germany, version 1.7.4).

### 4.9. Competition-Assay with hFX

Human coagulation factor X (hFX) competition assays on SKOV-3 cells using heparin (5 I.U.)/MgCl_2_ (5 µmol) treated HAdV50. The preparation of HAdV50 was performed as Zhang et al. described [26] and diluted to 1000 vpc. SKOV3 cells were cultured with McCoy’s 5 A medium (PAN-Biotech GmbH, Aidenbach, Germany) including 10% FCS (PAN-Biotech GmbH, Aidenbach, Germany) and 1% penicillin/streptomycin (PAN-Biotech GmbH, Aidenbach, Germany). After confluency cells were counted and plated in 96-well plates at a density of 2 × 10^4^. Several experimental situations and controls were compared in this study. HAdV50 without any treatment was used as a control. In the next group, the virus was mixed with hFX (8 μg per 1 mL, Haematologic Technologies, Essex, VT, USA) and added to the cells. In the third group, hFX was added after mixing the virus with heparin (5 I.U.) and magnesium chloride (5 µmol) and incubating for 15 min at room temperature. In the last arrangement, the virus was incubated with hFX for 15 min at room temperature and the mixture of heparin and MgCl_2_ was added. In each test setup, cells were incubated for 5 h at 37 °C in 200 μL serum-free medium, then medium was removed and cells were washed once with PBS. The medium was changed to McCoy’s 5 A medium containing 10% FBS and the cells were incubated for 72 h before luciferase measurement was performed. All experiments were performed in triplicates and repeated three times. For statistical analyses results were normalized to positive control, afterwards, a one-way ANOVA was performed.

### 4.10. Receptor Usage of HAdV5 on Treated CAR and CD46 Receptor Positive Cells

CHO-CAR cells stably encoding coxsackie adenovirus receptor (CAR) and CHO-C2 cells stably encoding CD46 subtype C2 receptor, were cultured in DMEM (PAN-Biotech GmbH, Aidenbach, Germany) including 10% FCS (PAN-Biotech GmbH, Aidenbach, Germany), 1% penicillin/streptomycin (PAN-Biotech GmbH, Aidenbach, Germany) and 1% non-essential amino acids (NEAA, (PAN-Biotech GmbH, Aidenbach, Germany). Additionally, both cell lines were selected with G418 (500µg/mL ThermoFisher, Waltham, MA, USA). Cells were counted and seeded at a density of 3 × 10^4^ per well in 96-well tissue culture plates in triplicates for each test. Cells were infected with HAdV5 (3 × 10^8^ virus particles) in the presence of heparin (5 I.U) and MgCl_2_ (5 µmol). A 26 h post-transduction a luciferase assay was performed as described before.

### 4.11. ELISA with IVIG and HAdV5 Treated with Modified Heparin

For ELISA experiments, 96-well plates were used and reagents were coated with 0.2 M Na_2_CO_3_/NaHCO_3_ (pH 9.5) overnight at 4 °C. HAdV5 (3 × 10⁶ virus particles) were incubated with heparin (5 I.U.) and MgCl_2_ (5 µmol) and applied to 96-well plate. As positive control wells were coated solely with virus. As controls for unspecific binding affinities, coating buffer only or heparin and MgCl_2_ were used. Subsequently, plates were washed two times with washing buffer (DPBS containing 0.5% Tween 20) and then blocked with blocking buffer (DPBS with 5% BSA) for 45 min at room temperature. After following five washing steps, IVIG (intravenous immunoglobulin, pooled and concentrated human IgGs, Privigen, Hattersheim am Main, Germany), pre-diluted 1:400 was added. IVIG was then successively diluted 1:2 in blocking buffer in 11 serial dilution steps. The plate was incubated at 37 °C for one hour. After 5 times washing, we added diluted second antibody (Goat pAb Hum IgG (HRP), Abcam, Cambridge, UK) 1:2000 in blocking buffer and added it to the wells before incubating the plate at 37 °C in a wet chamber for 70 min Then the plate was washed five times again. After adding 100 µL substrate solution (SIGMAFAST™ OPD, St. Louis, MO, USA) to each well the plate was incubated in a dark chamber for 30 min. Finally, we added 100 µL H_2_SO_4_ per well to stop the reaction. The plate was measured with a TECAN ELISA Reader at a wavelength of 492 nm. The evaluation of ELISA results was carried out with the statistic software GraphPad Prism (Version 8.3.1, GraphPad Software, San Diego, CA, USA) and the curve fit evaluation was determined by the One Site—Total method. The dissociation constants (Kd) were determined and drawn as lines into the curves.

### 4.12. Annexin V/7-AAD and Bcl-2 Activation Staining

Vero cells were cultured in Dulbecco’s Modified Eagle’s Medium (DMEM, PAN-Biotech GmbH, Aidenbach, Germany) supplemented with 10% fetal bovine serum (FBS, PAN-Biotech GmbH, Aidenbach, Germany) and 1% penicillin/streptomycin (PAN-Biotech GmbH, Aidenbach, Germany). A total of 1 × 10^5^ cells were counted and seeded per well in a 24 well plate in 1 mL medium. After 24 h, cells were treated w/wo modified heparin or magnesium chloride and heparin singly. Vero cells were cultured for 24 h under a humidified atmosphere of 5% CO_2_ and 37 °C. For analyzing apoptosis and cell death Annexin V/7-AAD Assay was performed according to manufactures information (Muse Annexin V and Dead Cell Assay, Sigma-Aldrich, St. Louis, MO, USA). The activation of the anti-apoptotic protein Bcl-2 was performed according to the manufacturer’s instructions (Muse Bcl-2 Activation Dual Detection Kit, Sigma-Aldrich, St. Louis, MO, USA). All experiments were performed in triplicates and the evaluation and graphical representation were carried out with GraphPad in (Version 8.3.1, San Diego, CA, USA).

## Data Availability

The data to this study can be shared upon reasonable request from the corresponding author.

## Supplementary Materials

The following are available online at https://www.mdpi.com/article/10.3390/ijms221810075/s1. Figure S1: Structure (inset) and chemical shift assignment of the repeating disaccharide unit of heparin using DQF-COSY spectrum acquired in 99.95% D_2_O at 37 °C, Figure S2: Further optimization of transduction conditions and transduction of human cells, Figure S3: Inhibitory effect of modified heparin on transduction rates of human adenovirus on human HeLa, Figure S4: Human coagulation factor X (hFX) competition assays and receptor usage of HAdV of treatment with MgCl_2_-modified heparin, Table S1: Experimental and calculated ^3^*J*_HH_ couplings of the iduronic acid residue of heparin, Supplementary Materials and methods.
