# Supplementary material for: Enhanced Antiviral Function of Magnesium Chloride-Modified Heparin on a Broad Spectrum of Viruses
Source: Int J Mol Sci. 2021 Sep 17;22(18):10075. doi: 10.3390/ijms221810075 (PMC8466540; doi:10.3390/ijms221810075)
Supplement: Supplementary file 1 [file ijms-22-10075-s001.zip › ijms-1368989-supplementary.pdf]

International Journal of Molecular Sciences

Supplemental Information

Table S1
Figures S1-S4

Enhanced Antiviral Function of Magnesium Chloride-Modified Heparin on a Broad Spectrum of Viruses Including SARS-CoV-2

Kemal Mese[1#], Oskar Bunz[1, 9#], Wolfram Volkwein[2], Sahithya P. B. Vemulapalli[7], Wenli Zhang[1], Sebastian Schellhorn[1], Kristin Heenemann[3], Antje Rueckner[3], Andreas Sing[2], Thomas W. Vahlenkamp[3], Anna-Lena Severing[4], Jian Gao[1], Malik Aydin[8], Dominik Jung[6], Hagen S. Bachmann[6], Kurt S. Zänker[5], Ulrich Busch[2], Armin Baiker[2§], Christian Griesinger[7*§], Anja Ehrhardt[1*§]

Table S1: Experimental and calculated $^3J_{HH}$ couplings of iduronic acid residue of heparin.

Proton pair	Experimental $^3J_{HH}$ (Hz)[a]			Calculated $^3J_{HH}$ (Hz)[b]	
	Heparin	Heparin-Mg^{2+}	Heparin-Ca^{2+}	Iduronic acid ($^1C_4$)	Iduronic acid (^2S_O)
I1-I2	3.4	3.2	2.7	1.6	6.7
I2-I3	5.8	5.6	4.5	3.0	11.1
I4-I5	2.9	2.9	2.1	1.7	6.5

[a]Derived from differences and sums of traces from 2D-DQF-COSY and NOESY spectra, [b]NMR=(giao,spin-spin), mpw1pw91/6-311++g(2d,p), scrf=(iefpcm,solvent=water).

Supplementary Figure S1

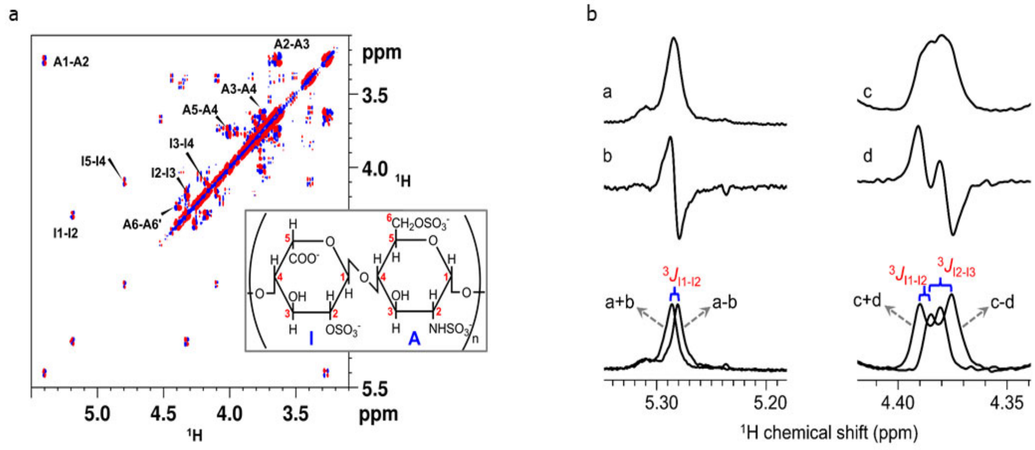

Figure S1: a, Structure (inset) and chemical shift assignment of the repeating disaccharide unit of heparin using DQF-COSY spectrum acquired in 99.95% D_2O at 37 °C. **b**, DISCONOE procedure for extracting the accurate $^3J_{HH}$ couplings from the broad ^{1}H signals. Traces from 2D-NOESY (a) and 2D-DQF-COSY (b) spectra corresponding to I1-I2 cross peaks of iduronic acid of heparin-Mg^{2+}. The difference (a-b) and sum (a+b) of above traces result in pseudo 1D spectra with in-phase doublet of respective proton pair. Similar procedure was carried out using the traces selected through I2 proton of iduronic acid lead to an in-phase doublet of doublets with $^3J_{I1\text{-}I2}$ and $^3J_{I2\text{-}I3}$ couplings as shown above. The $^3J_{I1\text{-}I2}$ and $^3J_{I2\text{-}I3}$ couplings obtained from the above mentioned procedure are 3.2 and 5.6 Hz for the iduronic acid residue of heparin-Mg^{2+}.

Supplementary Figure S2

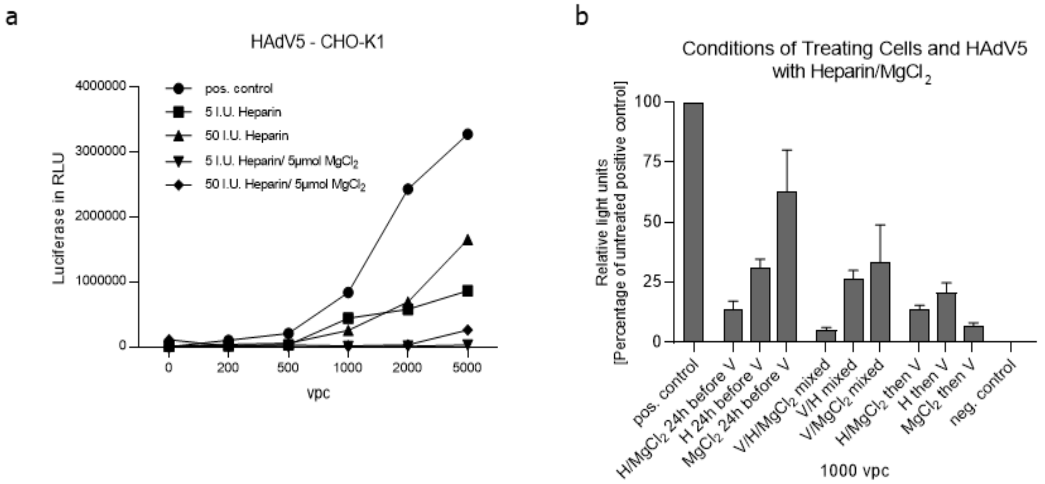

Figure S2: Further optimization of transduction conditions and transduction of human cells. **a**, Effects of 5 I.U. heparin and increasing virus dosages (vpc 200, 500, 1000, 2000, 5000) on HAdV5 infectivity in CHO-K1 cells. Experiments were performed with two different concentrations of heparin (5 or 50 I.U. per well), while the $MgCl_2$ concentration was kept stable at 5 µmol per well. Luciferase measurements were conducted 26 hrs post- transduction and expressed as relative light units (RLU). **b**, In-depth analyses of different HAdV5 transduction and incubation conditions with heparin (H) and $MgCl_2$. The different test series were performed with different combinations (heparin, $MgCl_2$, virus) and timings. As positive control (pos. control), CHO-K1 cells were infected with HAdV5 at 1000 vpc and the negative control (neg. control) remained uninfected. Heparin was applied at 5 I.U. and $MgCl_2$ at 5 µmol per well in a total volume of 100 µl. 26hrs after transduction a luciferase measurement was performed. The positive control was set to 100% and for all other experimental groups the percentage in correlation to the positive control was calculated. Data points represent mean standard error out of three independent experiments performed in triplicates.

Supplementary Figure S3

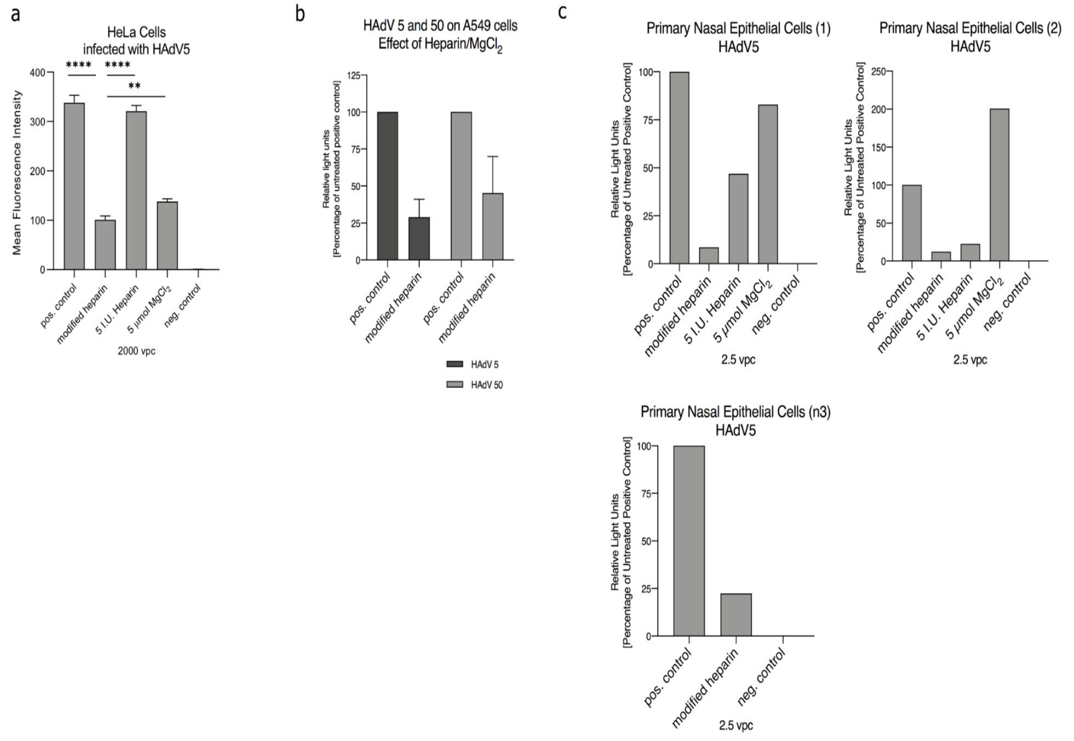

Figure S3: Inhibitory effect of modified heparin on transduction rates of human adenovirus on human HeLa, A549 and primary nasal epithelial cells. Luciferase and GFP measurements by flow cytometry were performed 26 hrs post- transduction. **a**, HeLa cells were infected with 2000 viral particles per cell (vpc) of HAdV5 and mean fluorescent intensity (MFI) was measured. **b**, Human A549 cells were infected with HAdV5 and HAdV50 using 2000 vpc and luciferase expression was analyzed 26 hrs post- transduction. **c**, Primary human epithelial cells were infected with treated and untreated HAdV5 using 2.5 vpc and luciferase expression was analyzed 26 hrs post-transduction. Three independent experiments (1-3) in primary nasal epithelial cells are shown and the mean of the technical replicates (n) is displayed. For experiments performed in HeLa and A549 cells, three independent experiments were performed in triplicates. Data points represent mean standard error. **** p-values ≤ 0.00005; ** p-values ≤ 0.005; * p-values ≤ 0.05.

Supplementary Figure S4

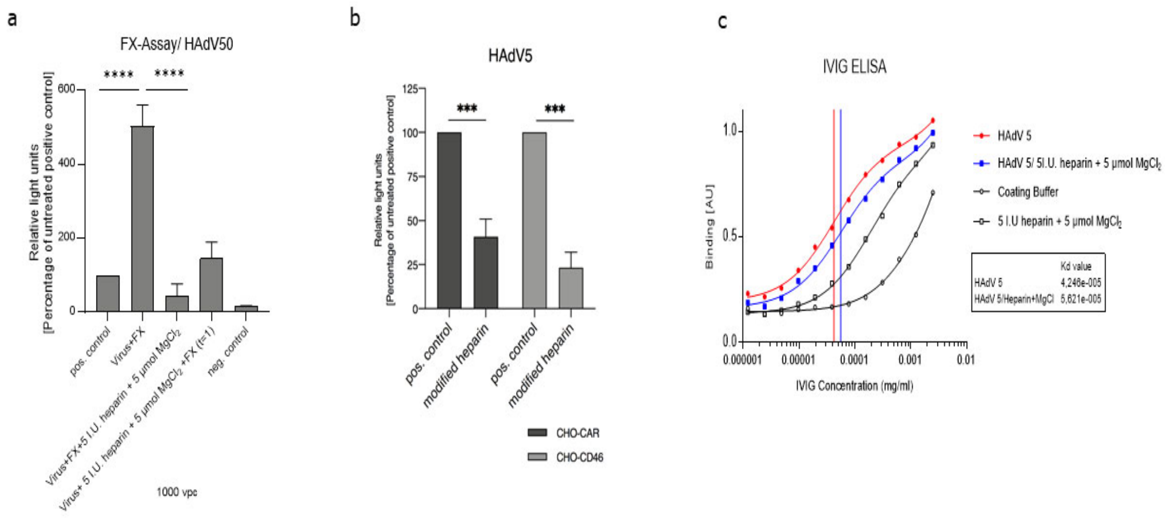

Figure S4: Human coagulation factor X (hFX) competition assays and receptor usage of HAdV of treatment with $MgCl_2$-modified heparin. **a,** Human coagulation factor X (hFX) competition assays with SKOV3 cells with modified heparin treated HAdV50. The first bar shows cells incubated with virus as positive control and the fourth bar shows the negative control referring to untreated cells. The second bar shows the results of HAdV50 and hFX only. For the third bar, virus, hFX and modified heparin were simultaneously applied to the cells and in the fourth bar cells were pre-incubated with virus and subsequently incubated with modified heparin and hFX for one hour. Experiments performed in triplicates are shown. For statistical analyses one-way ANOVA was performed, displayed are means + standard deviation. **** p-values ≤ 0.00005. **b**, Modified heparin (5 I.U. heparin + 5 µmol $MgCl_2$) treated HAdV5 shows a decreased luciferase expression on CHO-CAR and CHO-CD46 cells compared to the untreated virus (pos. control). Experiments were performed in triplicates and repeated three times. Statistical differences were examined by unpaired two-tailed t-test (*** p-values ≤ 0.0005). **c**, ELISA experiments to measure the binding of IVIG to modified heparin-coated adenovirus. As samples untreated HAdV5 and modified heparin treated HAdV5 were used. As controls coating buffer and modified heparin were applied. The nonlinear curve fit evaluation was determined by One Site – Total method. The dissociation constants (K_d) were analyzed and drawn into curves.
